# Phosphorus Reduces Negative Effects of Nitrogen Addition on Soil Microbial Communities and Functions

**DOI:** 10.3390/microorganisms8111828

**Published:** 2020-11-20

**Authors:** Zongwei Xia, Jingyi Yang, Changpeng Sang, Xu Wang, Lifei Sun, Ping Jiang, Chao Wang, Edith Bai

**Affiliations:** 1 CAS Key Laboratory of Forest Ecology and Management, Institute of Applied Ecology, Chinese Academy of Sciences, Shenyang 110016, China; xiazongwei@iae.ac.cn (Z.X.); jyyang17@126.com (J.Y.); sangcp1991@163.com (C.S.); wangxu113@mails.ucas.ac.cn (X.W.); sunlifei@iae.ac.cn (L.S.); jiangp@iae.ac.cn (P.J.); baie@iae.ac.cn (E.B.); 2 University of Chinese Academy of Sciences, Beijing 100049, China; 3 Key Laboratory of Geographical Processes and Ecological Security in Changbai Mountains, Ministry of Education, School of Geographical Sciences, Northeast Normal University, Changchun 130024, China

**Keywords:** nitrogen deposition, phosphorus addition, microbial community, functional potential, temperate forest, high throughput sequencing

## Abstract

Increased soil nitrogen (N) from atmospheric N deposition could change microbial communities and functions. However, the underlying mechanisms and whether soil phosphorus (P) status are responsible for these changes still have not been well explained. Here, we investigated the effects of N and P additions on soil bacterial and fungal communities and predicted their functional compositions in a temperate forest. We found that N addition significantly decreased soil bacterial diversity in the organic (O) horizon, but tended to increase bacterial diversity in the mineral (A) horizon soil. P addition alone did not significantly change soil bacterial diversity but mitigated the negative effect of N addition on bacterial diversity in the O horizon. Neither N addition nor P addition significantly influenced soil fungal diversity. Changes in soil microbial community composition under N and P additions were mainly due to the shifts in soil pH and NO_3_^−^ contents. N addition can affect bacterial functional potentials, such as ureolysis, N fixation, respiration, decomposition of organic matter processes, and fungal guilds, such as pathogen, saprotroph, and mycorrhizal fungi, by which more C probably was lost in O horizon soil under increased N deposition. However, P addition can alleviate or switch the effects of increased N deposition on the microbial functional potentials in O horizon soil and may even be a benefit for more C sequestration in A horizon soil. Our results highlight the different responses of microorganisms to N and P additions between O and A horizons and provides an important insight for predicting the changes in forest C storage status under increasing N deposition in the future.

## 1. Introduction

The atmospheric nitrogen (N) deposition rate has been increasing because of anthropogenic activities [1,2], which raises serious negative ecological consequences, such as soil acidification, plant productivity decline, biodiversity loss, and soil nutrient imbalance [3,4,5,6,7]. Microorganisms are widespread in soil and play an important role in the ecosystem functions involved in nutrient cycling, organic matter decomposition, and greenhouse gas production [8,9,10]. Thus, the changes in soil microbial biomass, community structure and lifestyle induced by increasing N deposition may influence microbial functions and ultimately have feedback on ecosystem C and N dynamics [11,12].

Some studies have been made to assess the responses of soil microbial biomass to N deposition, and they found N deposition generally suppresses microbial biomass [13,14]. The main reason may be that N deposition could reduce soil base cations and induce soil acidification [15]. More importantly, the increasing N deposition can change microbial diveristy [16,17] and community composition [18,19]. The oligotrophic-copiotrophic theory suggests that under N addition copiotrophic bacterial taxa can grow rapidly and are dominant in nutrient-rich soil while oligotrophic groups grow slowly and are enriched in nutrient-limited soil [20,21]. However, many microbes show overlaps in trophic modes and ecological functions [22], and their responses to increasing N deposition are related not only to soil nutrient status but also to the changes in other soil conditions (such as pH, mineral cations) [15]. However, the relative significance of N availability (soil NH_4_^+^-N and NO_3_^−^-N) and soil acidification in determining N enrichment-induced changes in soil microbial community is still unclear [23]. This may depend on the rates of N deposition as well as the initial N conditions in investigated ecosystems [24]. Moreover, increasing N deposition also impacts the plant community diversity and primary production, which both are assumed to impact the C allocation to soils via above- and below-ground plant biomass [25], and thus indirectly affect soil microorganisms [26,27].

In general, tropical and subtropical forests are often limited by phosphorus (P) [28], while temperate and the boreal forest ecosystems are limited by N [29]. However, the P availability in temperate forest soil may be greatly limited when increased N deposition excesses the threshold of an ecosystem [30]. It has been reported that N addition could decrease the soil inorganic P availability because of the changes in both microbial properties and plant P uptake [31]. Whether P addition can alter the effects of increasing N deposition on the soil microbial communities and functional potentials and therefore the C and N cycling in the temperate forest remains uncertain. The current N deposition rate in the Changbai mountain region in Jilin province of China is ~27 kg N ha^−1^ yr^−1^ [32]. Although this rate approaches the critical load of nutrient N deposition in this region and tends to continuously increase in the future [32,33], the temperate broad-leaved Korean pine forest in this region has been considered as an N-limited ecosystem. We build an N and P additions experiment in a typical broad-leaved Korean pine forest to study the ecological effects of the N addition and their interaction with P addition on soil microbes. Our main research questions were: (1) Does the increased N deposition influence the soil bacterial and fungal communities and their functional potentials in the temperate forest, and what factors explain these changes (soil pH or N availability)?; (2) Does the P addition alleviate the N deposition effects on soil microorganisms or interact with N deposition to affect the soil microorganisms?; (3) Are responses of soil microbial communities to N and P addition between organic soil (O horizon) and mineral soil (A horizon) different? The understanding of the effects of N and P addition on the soil microbial community can help us to better predict how the soil microbial activities will respond to environmental change and therefore soil C and N cycling mediated by them in temperate forest ecosystems of China.

## 2. Materials and Methods

### 2.1. Experimental Setup and Soil Sampling

A temperate broad-leaved Korean pine forest was selected for the long-term experimental field located at Changbai Mountains in Jilin province China (41°42′ N, 127°38′ E). This region is a typically temperate climate with a mean annual temperature of 3.6 °C and a mean annual precipitation of 745 mm. The dominant plant species include *Pinus koraiensis*, *Larix olgensis*, *Abies nephrolepis*, *Quercus mongolica*, *Acer mono*, *Fraxinus mandshurica*, *Tilia amurensis*, and *Betula costata*. The soil is classified as dark brown soil developed from volcanic ash (Albic Luvisol) [34].

The N and P addition experiment was established in May 2015. Three study plots (20 × 20 m each) were selected for control (C, without N and P addition), nitrogen addition (N), phosphorus addition (P), and nitrogen with phosphorus addition (NP) treatments, respectively. NH_4_NO_3_ and NaH_2_PO_4_ were added to the surface of the plot floor in water solution for N, P, and NP addition treatments at the beginning of each month from May to September. The application rates of N and P were 5 g N m^−2^ yr^−1^ and 2.5 g P m^−2^ yr^−1^, respectively.

Soil from O horizon (0–5 cm) and A horizon (5–15 cm) were sampled after removing litters in September 2018. Soil samples were sieved (2 mm) and thoroughly homogenized. Each soil sample was split into two subsamples. One subsample was stored at −20 °C until DNA extraction, and another was stored at 4 °C for soil chemical analyses.

### 2.2. Soil Chemical Analyses

Soil pH was determined by a pH meter (Leici, Shanghai, China) in the liquid after shaking soil-water (1:2.5 *w*/*v*) mixture for 30 min. Soil organic carbon (SOC) and total nitrogen (TN) were determined in an elemental analyzer (vario MACRO cube, Elementar, Hanau, Germany). Soil NH_4_^+^ and NO_3_^−^ were extracted with 2 M KCL solution, and their contents were measured by a flow injection analyzer (Futura, Alliance, Frépillon, France). Soil total phosphorus (TP) was analyzed as previously described methods [35]. Soil microbial biomass carbon (MBC), nitrogen (MBN) and phosphorus (MBP) were measured by the chloroform fumigation method [36,37,38]. MBC, MBN, and MBP were calculated by subtracting total C, N, and P in extracts of the non-fumigated subsamples from the fumigated subsamples by using a conversion factor of 0.45 for MBC, 0.54 for MBN, and 0.4 for MBP, respectively [36,39,40].

### 2.3. Soil DNA Extraction, Sequencing and Data Processing

Soil DNA was extracted from 250 mg freeze-dried soil using a Mobio PowerSoil DNA Isolation Kit (MoBio Laboratories, Carlsbad, CA) according to the manufacturer’s instructions. The quantity and quality of extracted DNA were estimated by a NanoDrop Spectrophotometer (Thermo Scientific, Waltham, MA, USA).

The primer pairs 515F(5′- GTG CCA GCM GCC GCG GTA A -3′)/806R(5′- GGA CTA CHV GGG TWT CTA AT -3′) and ITS1(5′- CTT GGT CAT TTA GAG GAA GTA A -3′)/ITS2(5′- TGC GTT CTT CAT CGA TGC-3′) with 8-bp barcodes at the 5′-end of them were used to amplify the V4-V5 region of the 16S rRNA genes of bacteria, and the ITS1 region of the ITS genes of fungi, respectively. PCR amplification was conducted in triplicate 25 μL mixtures, which contained 12.5 μL of 2×Taq Plus Master Mix, 1 μL of 5 μM of each primer, 3 μL of 2 ng μL^−1^ BSA, 30 ng of template DNA and ddH_2_O filled to 25 μL. The high-throughput sequencing was performed on an Illumina Miseq system (Illumina, San Diego, CA, USA) at Allwegene technology company (Beijing, China).

The raw data were qualified through screening. Sequences were removed if they were shorter than 200-bp, had a low-quality score (≤20), contained ambiguous bases or did not exactly match to primer sequences and barcode tags. Qualified reads were assigned to each sample according to the sample-specific barcode sequences which were trimmed together with primers using Illumina Analysis Pipeline Version 2.6. And then the dataset was analyzed using VSEARCH (version 2.7.1) [41] and QIIME (Version 1.8.0) [42]. The sequences were clustered into operational taxonomic units (OTUs) at a similarity level of 97% by UPARSE method [43]. Two rarefied OTU tables (53,209 and 35,067 reads per sample for bacteria and fungi, respectively) were used for the downstream analyses. The Chao1, observed species, phylogenetic diversity and Shannon index were calculated to estimate the microbial alpha-diversity. The Ribosomal Database Project (RDP) Classifier tool [44] was used to classify all sequences into different taxonomic groups based on the SILVA database [45] and Greengenes database [46] for bacterial 16S rRNA and UNITE database [47] for fungi ITS, respectively. Bacterial and fungal potential function categories were predicted according to FAPROTAX [48] and FUNGuild [49] databases, respectively. The principle of functional prediction is searching against FAPROTAX or FUNGuild databases (connecting taxonomy with function) and converting taxonomic microbial community compositions (e.g., in the form of an OTU table) into putative functional compositions, based on taxa identified in a sample. It should be noted that we filtered predicted guilds of fungi based on the confidence ranking (three likelihood levels: “highly probable”, “probable”, and “possible”) and only guilds with the confidence ranking of “highly probable” and “probable” were used for subsequent analysis. All the raw sequence reads have been submitted to the Sequence Read Achieve (SRA) database of NCBI and were retrievable under the accession number PRJNA643350.

### 2.4. Statistics

Two-way ANOVA followed by Duncan’s multiple-comparison test was used to analyze the effects of N, P, and NP addition treatments on soil characteristics, indices of community diversity, abundances of dominant taxa, and also predicted functional categories. Pearson′s rank correlation and regression analyses were performed between soil characteristics and individual phyla, between soil characteristics and indices of community diversity and composition, and between soil characteristics and functional genes’ abundance predicted. The correlation between microbial community structure and soil variables as determined by the Mantel test with Pearson’s correlation coefficient and 999 permutations. A redundancy analysis (RDA) was performed to identify soil characteristics that primarily account for the variation of microbial community composition in N, P and NP addition treatments. Permutational multivariate analyses of variance (PERMANOVA) were conducted to test the effects of different N, P, and NP addition treatments on microbial community composition and predicted function structure. All statistical analyses were performed in SPSS 17.0 (SPSS Inc., Chicago, IL, USA) for windows or in the program R (version.3.4.0) [50].

## 3. Results

### 3.1. Soil Characteristics under N and P Addition

Soil pH and NH_4_^+^-N decreased significantly due to N, P and NP additions in soil O horizon (Table S1), but soil NO_3_^−^-N and inorganic N (NH_4_^+^-N + NO_3_^−^-N) increased. TN and TP did not change under all treatments in O horizon. P addition depressed MBC and MBN, but did not affect MBP in O horizon (Table S1, Duncan’s multiple range test). Moreover, N and P additions had an interactive effect on soil pH, NH_4_^+^-N, TN, MBC, MBN, and MBP (Supplementary Table S2, two-way ANOVA *p* < 0.05).

In A horizon, soil pH was significantly decreased under P addition. Soil NO_3_^−^-N and inorganic N were generally enhanced by all addition treatments, while NH_4_^+^-N showed a decreasing trend (Table S1). All addition treatments showed positive effects on MBC and MBP. Moreover, NP addition also significantly increased soil SOC, TN, and MBN (Supplementary Table S1, Duncan’s multiple range test). However, N and P had only interactive effects on soil pH, NO_3_^−^-N, and MBN (Supplementary Table S2, two-way ANOVA *p* < 0.05).

### 3.2. Microbial Diversity and Community Composition under N and P Addition

**Figure 1:**
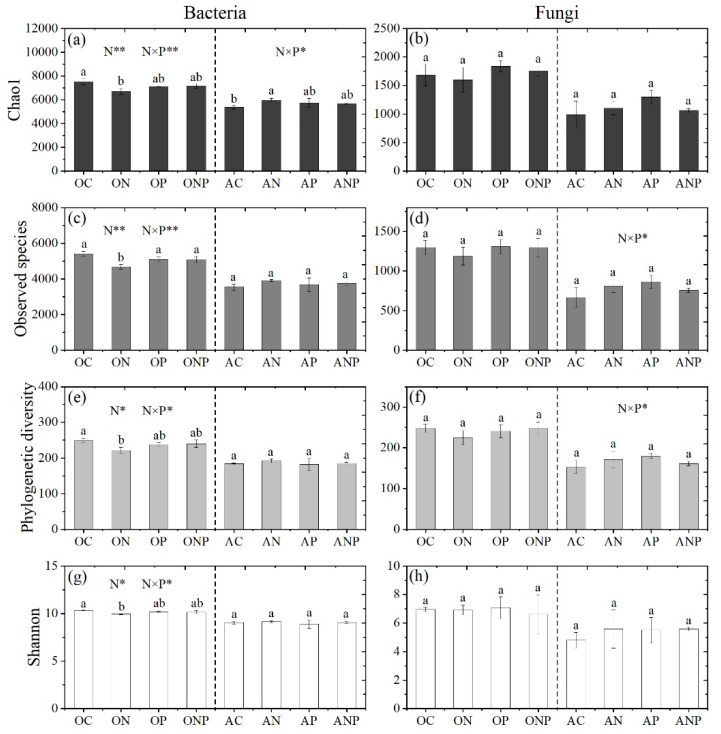
Soil bacterial (**a**,**c**,**e**,**g**) and fungal (**b**,**d**,**f**,**h**) diversity index (Chao1 (**a**,**b**), Observed species (**c**,**d**), Phylogenetic diversity (**e**,**f**) and Shannon (**g**,**h**) under N and P additions. Different letters above the bars indicate significant differences (one-way ANOVA, *p* < 0.05, Duncan’s multiple range test) among different fertilization addition treatments. N, P and N × P showed the effects of N, P and NP interaction on bacterial and fungal diversities, respectively (two-way ANOVA, * *p* < 0.05, ** *p* < 0.01). OC, ON, OP and ONP indicate the O horizon soils (0–5 cm) under control (C, without N and P addition), N addition, P addition and NP addition, respectively. AC, AN, AP and ANP indicate the A horizon soils (5–15 cm) under control (C, without N and P addition), N addition, P addition and NP addition, respectively.

N addition greatly reduced bacterial α-diversity in O horizon soil (*p* < 0.05, Duncan’s multiple range test), while P and NP additions had no impact on bacterial α-diversity (Figure 1). Additionally, N and P had interactive effects on bacterial α-diversity in this horizon (Figure 1). In A horizon, bacterial Chao1 tended to be increased by all addition treatments and showed a significant response to N addition (Figure 1, *p* < 0.05, Duncan’s multiple range test). Moreover, all addition treatments did not change the fungal α-diversity in both horizons, but fungal α-diversity showed a decreasing trend by N addition in O horizon and positive responses to both N and P additions in A horizon (Figure 1).

Soil bacterial α-diversity was negatively correlated with soil NO_3_^−^-N and generally positively correlated with soil pH, NH_4_^+^-N and MBN in O horizon soil (Table 1). Moreover, observed species of bacteria and fungi were negatively correlated with pH, NH_4_^+^-N in A horizon, while Chao1 and Shannon of bacteria and fungi were also negatively correlated with pH and NH_4_^+^-N, respectively (Table 1).

**Figure 2:**
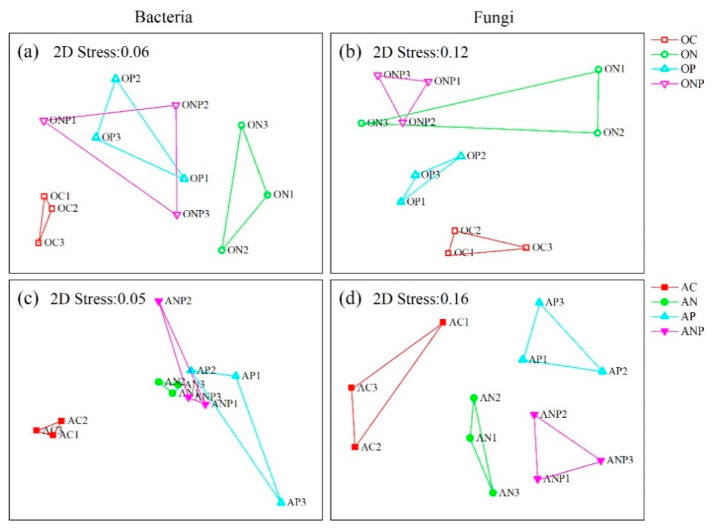
Non-metric multidimensional scaling (NMDS) plots of bacterial (**a**,**c**) and fungal (**b**,**d**) community compositions under N and P additions. OC, ON, OP and ONP indicate the O horizon soils (0–5 cm, (**a**,**b**)) under control (C, without N and P addition), N addition, P addition and NP addition, respectively. AC, AN, AP and ANP indicate the A horizon soils (5–15 cm, (**c**,**d**)) under control (C, without N and P addition), N addition, P addition and NP addition, respectively.

The NMDS analyses showed that the N, P, and NP additions shifted the bacterial community composition towards the same direction along *X*-axis in both horizons (Figure 2a,c), and two-way PERMANOVA analysis indicated that N addition had a larger influence on the variation of bacterial community composition in O horizon and P addition did that in A horizon (Table 2).

**Figure 3:**
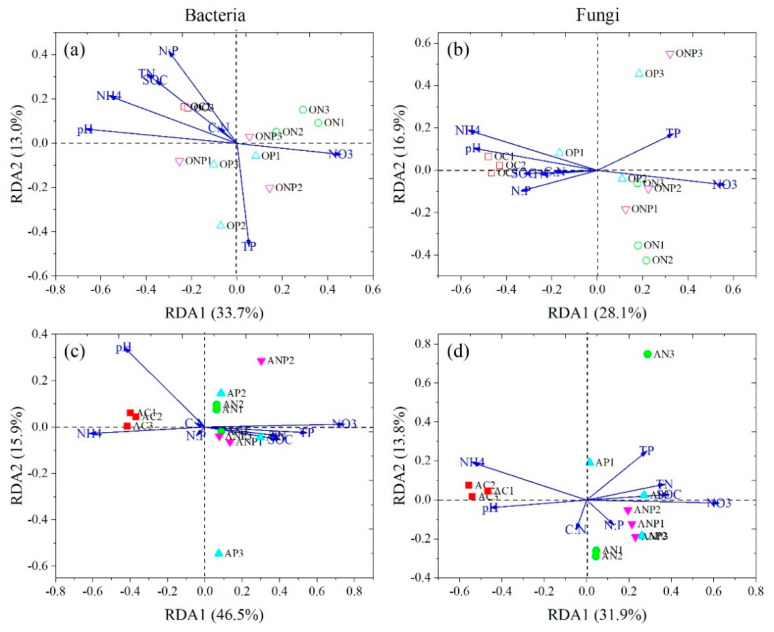
Redundancy analysis (RDA) of the bacterial (**a**,**c**) and fungal (**b**,**d**) community compositions and soil variables. OC, ON, OP and ONP indicate the O horizon soils (0–5 cm, (**a**,**b**)) under control (C, without N and P addition), N addition, P addition and NP addition, respectively. AC, AN, AP and ANP indicate the A horizon soils (5–15 cm, (**c**,**d**)) under control (C, without N and P addition), N addition, P addition and NP addition, respectively.

All addition treatments also changed fungal community compositions in the same direction in A horizon (Figure 2d). However, the fungal community compositions were differentiated to opposite directions by N and P additions in O horizon (Figure 2b). The two-way PERMANOVA revealed a significant interaction effect of N and P additions on changing the fungal community compositions in A horizon (Table 2). Further, the RDA analysis and mantel test showed that soil pH and NO_3_^−^-N well explained the variations in bacterial and fungal community compositions (Figure 3, Table 3).

### 3.3. Dominant Microbial Taxa under N and P Addition

**Figure 4:**
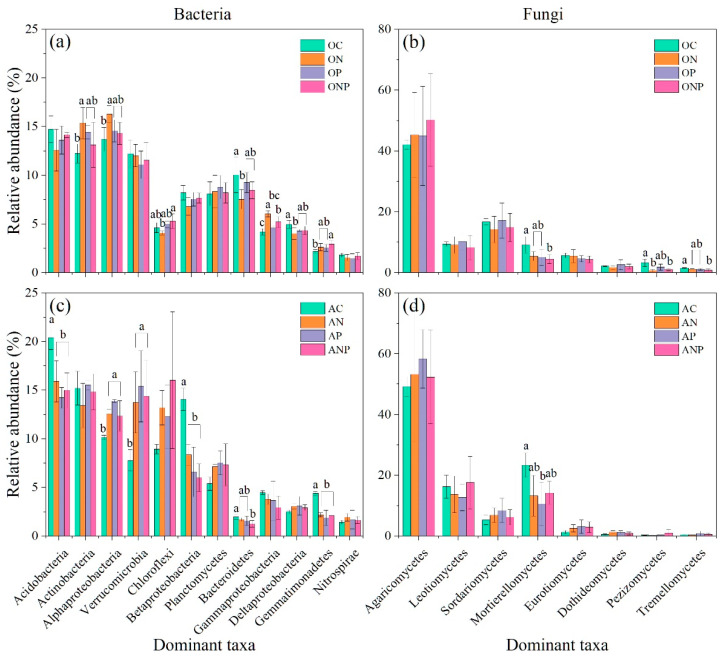
The relative abundance of dominant bacterial (**a**,**c**) and fungal (**b**,**d**) groups under N and P additions. Values are mean ± standard deviation (*n* = 3). Different letters above the bars indicate significant differences among different fertilization addition treatments (one-way ANOVA, *p* < 0.05, Duncan’s multiple range test). OC, ON, OP and ONP indicate the O horizon soils (0–5 cm, (**a**,**b**)) under control (C, without N and P addition), N addition, P addition and NP addition, respectively. AC, AN, AP and ANP indicate the A horizon soils (5–15 cm, (**c**,**d**)) under control (C, without N and P addition), N addition, P addition and NP addition, respectively.

The N addition increased the relative abundance of Actinobacteria, Alphaproteobacteria and Gammaproteobacteria, but decreased Bacteroidetes and Deltaproteobacteria in O horizon soil (Figure 4a). However, the P addition did not change the relative abundance of these observed bacterial taxa in O horizon. NP addition only increased the relative abundance of Gammaproteobacteria and Gemmatimonadetes (Figure 4a). Further, N, P, and NP addition increased Alphaproteobacteria and Verrucomicrobia, and decreased Acidobacteria, Betaproteobacteria, and Gemmatimonadetes in A horizon soil (Figure 4c). P addition played an important role in changing the relative abundance of bacterial taxa especially Acidobacteria, Alphaproteobacteria, Verrucomicrobia, Betaproteobacteria and Gemmatimonadetes (Supplementary Table S3). In addition, the interaction of N and P addition also showed significant effects on the relative abundance of Acidobacteria, Alphaproteobacteria, Betaproteobacteria, and Gemmatimonadetes in A horizon soil (Supplementary Table S3).

The N addition only decreased Pezizomycetes, and NP addition decreased Mortierellomycetes and Tremellomycetes in O horizon (Figure 4b). The P addition decreased Mortierellomycetes in A horizon (Figure 4d). Soil pH, NH_4_^+^-N, and NO_3_^−^-N generally showed significant correlation with relative abundances of dominant bacterial and fungal taxa (Supplementary Tables S4 and S5).

### 3.4. Microbial Functional Potentials under N and P Addition

**Figure 5:**
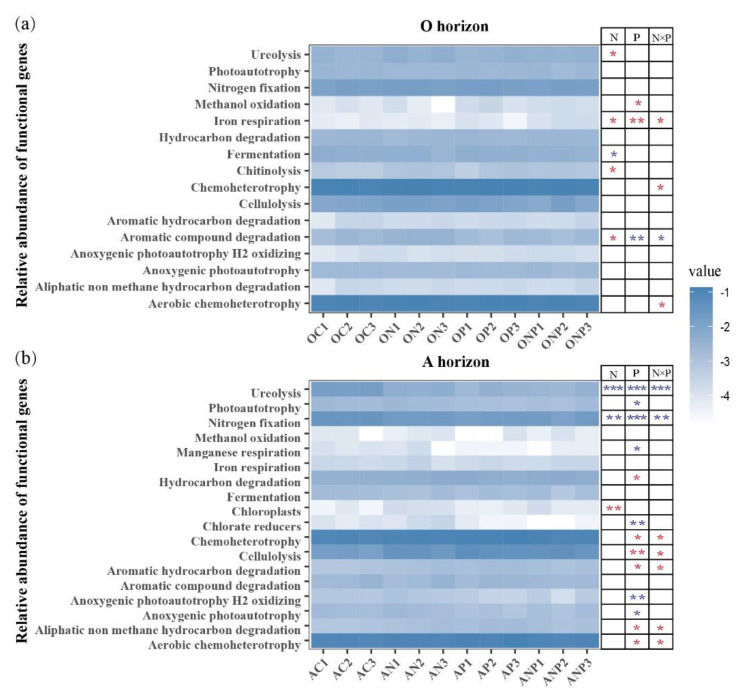
The relative abundance of putative bacterial functional categories under N and P additions based on the FAPROTAX database. N, P and N × P showed the effects of N, P and NP interaction on bacterial and fungal diversities, respectively (two-way ANOVA, * *p* < 0.05 ** *p* < 0.01 and *** *p* < 0.001). Red asterisks indicate positive effects and blue asterisks indicate negative effects. OC, ON, OP and ONP indicate the O horizon soils (0–5 cm, (**a**)) under control (C, without N and P addition), N addition, P addition and NP addition, respectively. AC, AN, AP and ANP indicate the A horizon soils (5–15 cm, (**b**)) under control (C, without N and P addition), N addition, P addition and NP addition, respectively.

A total of 63 functional categories were matched when linking the bacterial community to the FAPROTAX database. Two-way PERMANOVA analysis indicated that the overall effects of N and P addition on bacterial functional composition in O horizon were not significant (*p* > 0.05). But, there was a significant effect of P addition (*p* < 0.01) and also a slight effect of N addition (*p* = 0.076) on the bacterial functional composition in A horizon (Supplementary Table S6). Many bacterial functional groups related to C and N metabolic processes were influenced by N and P addition treatments (Figure 5 and Supplementary Table S7). We generally observed distinct functional groups affected by N and P addition between O and A horizons, and more functional groups in A horizon than O horizon were influenced (Figure 5). N addition significantly enhanced ureolysis, degradation of aromatic compound and chitinolysis processes and reduced fermentation in O horizon (Figure 5a and Supplementary Table S7), while nitrogen fixation and ureolysis functions were reduced but chloroplasts were elevated by N addition in A horizon soil (Figure 5b and Supplementary Table S7). P addition significantly enhanced chemoheterotrophy, aerobic chemoheterotrophy, cellulolysis, degradations of hydrocarbon, aromatic hydrocarbon, and aliphatic non methane hydrocarbon, while the reduced functional processes included ureolysis, nitrogen fixation, chlorate reducers, manganese respiration, anoxygenic photoautotrophy H_2_ oxidizing, photoautotrophy, and anoxygenic photoautotrophy (Figure 5b and Supplementary Table S7).

**Figure 6:**
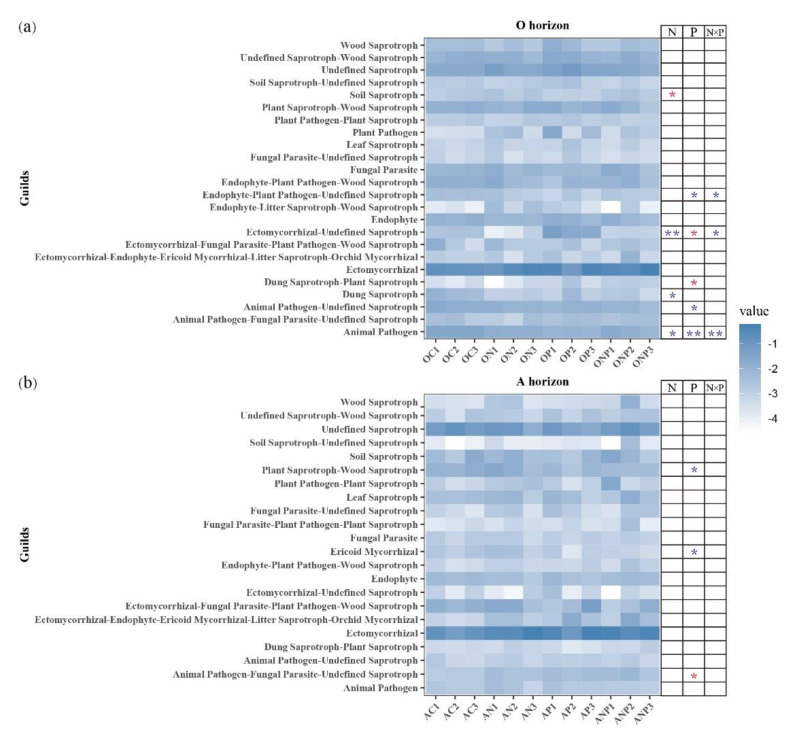
The relative abundance of putative fungal functional guilds under N and P additions based on the FUNGuild database. N, P and N × P showed the effects of N, P and NP interaction on bacterial and fungal diversities, respectively (two-way ANOVA, * *p* < 0.05, ** *p* < 0.01). Red asterisks indicate positive effects and blue asterisks indicate negative effects. OC, ON, OP and ONP indicate the O horizon soils (0–5 cm, (**a**)) under control (C, without N and P addition), N addition, P addition and NP addition, respectively. AC, AN, AP and ANP indicate the A horizon soils (5–15 cm, (**b**)) under control (C, without N and P addition), N addition, P addition and NP addition, respectively.

We found 35 guilds from the FUNGuild database when predicting the fungal functional potentials. N addition significantly decreased dung saprotroph (Schizothecium), animal pathogen (Metarhizium, Pochonia, Lecanicillium), ectomycorrhizal-undefined saprotroph (Thelephoraceae), and increased soil saprotroph (Geomyces) in O horizon (Figure 6a). P addition tended to reduce endophyte-plant pathogen-undefined saprotroph (Pezicula), animal pathogen-undefined saprotroph (Exophiala) and animal pathogen (Metarhizium, Pochonia, Lecanicillium), and increase ectomycorrhizal-undefined saprotroph (Thelephoraceae), and dung saprotroph-plant saprotroph (Sordariaceae) in O horizon. There were interaction effects of N × P on decreasing endophyte-plant pathogen-undefined saprotroph (Pezicula), and decreasing animal pathogen (Metarhizium, Pochonia, Lecanicillium) (Figure 6a). However, there were opposite effects for N addition and P addition on ectomycorrhizal-undefined saprotroph (Thelephoraceae) in O horizon (Figure 6a and Table S8). Generally, only P addition had significant effects on plant saprotroph-wood saprotroph (Hyaloscyphaceae), ericoid mycorrhizal (Oidiodendron), and animal pathogen-fungal parasite-undefined saprotroph (Herpotrichiellaceae) in A horizon (Figure 6b).

## 4. Discussion

### 4.1. Microbial Diversity and Biomass under N and P Addition

We found N addition decreased bacterial diversity in O horizon soil, while it tended to increase the bacterial diversity in A horizon (Figure 1). This contrast response of soil bacterial diversity between O and A horizons to increasing N deposition was largely dependent on soil acidification status and background nutrient availability [16]. It has been reported that soil bacterial diversity was positively correlated with soil pH in acidic-neutral regions [51,52,53]. Despite increasing N availability (Table S1), N addition caused severe soil acidification (about 0.8 pH units, Supplementary Table S1) in O horizon soil in this study, which could directly decrease some acid-sensitive microbial communities. Moreover, acidification can decrease the availabilities of soil base mineral cations such as calcium, magnesium [54] and accumulate toxic aluminum cation [33], which might indirectly influence the microbial communities [55]. These changes in soil pH and base mineral cations have negative effects on the survival of some bacterial species and may lead to the loss of rare species. This is supported by the sharply reduced numbers of rare bacterial OTUs under N addition compared to the control in O horizon soil (Supplementary Table S9). However, we found no changes in soil pH but increases in SOC and inorganic N under N addition in A horizon. This might satisfy the soil bacterial demands for C and N, and then stimulate the growth of most bacteria communities [56]. Consequently, the accumulation of microbial biomass and bacterial α-diversity increased (Figure 1, Supplementary Table S1). Therefore, the soil acidification induced by increasing N deposition controlled the bacterial diversity in O horizon, while increased C and N availabilities determined the bacterial diversity in A horizon. On the contrary, fungal diversity was not significantly changed by increasing N deposition (Figure 1). This may be explained by the fact that fungi and bacteria have different cell structures [57], by which fungi are more tolerant than bacteria to elevated H^+^ or even Al^3+^ ions due to soil acidification induced by increasing N deposition [58,59].

Further, our results indicated that the P addition may alleviate the negative effect of increasing N deposition on soil microbes. First, the decreased bacterial α-diversities (especially observed species) under N addition was restored by NP addition in O horizon (Figure 1). Second, soil microbial biomass was greatly promoted under NP addition in A horizon (Supplementary Table S1). This result was coincided with the studies from P-limited old-growth tropical ecosystems [60,61], but was different from a P-unlimited subtropical forest where increased soil P availability did not change microbial biomass [62]. It is possible that the N and P were limited in A horizon for microbes in this studied forest because either N or P addition increased microbial biomass (Supplementary Table S1).

### 4.2. Microbial Composition and Structure under N and P Addition

The relative abundance of dominant taxa showed different response patterns to N addition (Figure 4), which can be largely explained by their different ecological trophic strategies [20]. The copiotrophic bacterial taxa, such as Actinobacteria, Alpha- and Gammaproteobacteria, adopt r-strategy and can grow fast under nutrient-rich environment compared to oligotrophic taxa with k-strategy, such as Acidobacteria, Betaproteobacteria, and Gemmatimonadetes [20,21,63]. Though Verrucomicrobia was considered to be the oligotrophic taxa [64], it showed an unexpected increase by N addition in A horizon in this study. This result confirmed with findings obtained from an agricultural soil with 34-year N fertilizer in northeast China [65]. Likewise, Bacteroidetes have been described as copiotrophic bacteria [20,21], but showed a decrease by N addition (5 g N m^−2^ yr^−1^) in this study (Figure 4). Wu et al. [66] found that the relative abundance of Bacteroidetes was increased by high N addition treatment (24 g N m^−2^ yr^−1^) but showed a decreased trend at low N addition level (6 g N m^−2^ yr^−1^). Also, the relative abundances of Bacteroidetes and Verrucomicrobia showed no response to all N addition levels (three N-addition levels: 5, 10, and 15 g N m^−2^ yr^−1^) in a natural steppe system [67]. Thus, these results suggested that different taxa may have diverse response thresholds to N addition among different ecosystems, and how they adapt to the N addition may depend on original soil nutritional contexts.

Soil fungi Mortierellomycetes, Pezizomycetes, and Tremellomycetes were found to decrease in O horizon under N addition in this study (Figure 4, Supplementary Table S3), which was partly in line with results from Morrow et al. [68]. This is probably because these fungal taxa are sensitive to soil pH (Supplementary Table S4). The changes in soil N availability under N addition may also influence their relative abundances, for their positive correlations with soil NH_4_^+^-N contents and negative with NO_3_^−^-N contents (Supplementary Table S4). Moreover, both RDA and mantel test results (Figure 3 and Table 3) demonstrated that soil acidification together with the changes in N availability (especially NO_3_^−^-N) could explain the shift in bacterial and fungal community compositions under increased N deposition in this temperate forest.

### 4.3. Microbial Functional Potentials under N and P Addition

The N addition increased the C cycling processes in O horizon (Figure 4), indicating the increased demand for the labile C resource to balance the N requirements and also to satisfy the high metabolic activity of soil microorganisms. These enhanced C cycling processes by N addition implied that the N addition stimulated the activity of soil microorganisms and accelerated the decomposition of soil organic matter and probably result in the loss of soil C by microbial respiration in O horizon soil. The P addition changed the positive effect of N addition alone to the negative effect of NP addition on the degradation of aromatic compound methanol oxidation (Figure 5a). Moreover, the NP integration eliminated the positive effects of N addition on chitinolysis. This suggested that P addition may alleviate the effects of N deposition on the soil microbial processes and alter soil C and N dynamics of forest topsoil. In contrast, the P addition not only accelerated the decomposition of soil organic matter but also elevated chemoheterotrophy process and suppressed respiration process in A horizon (Figure 5b), indicating that most of the products from decomposed compounds probably were used as the substrate for bacterial assimilation other than dissimilation, as evidenced by the increase in microbial biomass (Supplementary Table S1). Therefore, we speculate that increasing N deposition may lead to more C losses from O horizon soil, but P addition could eliminate it and even increase C immobilization in A horizon soil by regulating bacterial processes.

Although N and P additions had not significantly changed the whole fungal functional composition, several fungal function guilds were still changed. The soil saprotroph fungi (Geomyces), which were known to use cellulose as the food resource [69], were increased by N addition in O horizon suggesting a possible increase in the soil organic matter mineralization. However, the P addition can relieve the positive effects of N addition on the soil saprotroph fungi (Figure 6a), indicating the P addition under increasing N deposition can slow soil C loss in O horizon by suppressing soil saprotroph fungi. Additionally, the decrease of plant saprotroph-wood saprotroph fungi by P addition in A horizon soil (Figure 6b) implied that fungi mediated the decomposition of plant recalcitrant carbon likely be retarded and this may be a benefit for C sequestration [12]. Thereby, the P addition could mitigate or alter the effect of increasing N deposition on the soil saprotroph fungi and then organic matter decay mediated by these fungi. Overall, N and P additions showed negative effects on soil mycorrhizal fungi (Figure 6 and Supplementary Table S3), which was agreed with the results observed in temperate or tropical forests [70,71]. These negative responses of mycorrhizal fungi to N and P additions suggested that N and P additions elevated the soil N and P availability to plant and probably decreased mycorrhizal fungi N and P transfer to host plants [72,73]. The investment of host plants in mycorrhizal fungi may be decreased for optimal economic strategy, thus hyphae growth and production of mycorrhizal fungi were reduced correspondingly [74,75]. Therefore, increased N deposition and P addition partly weakened the symbiotic relationship between arbuscular mycorrhizal fungi and host plants [76]. It should be noted that the informations on functional groups of FAPROTAX and FUNGuild databases are established mostly based on the current literature [48,49], and they include limited culturable microbes with defined functions comparing to large and complex soil microbiome; therefore, further researches on functional genes, such as metagenomics sequencing [77] or functional genes’ quantification (Geochip) [78], would be helpful for better understanding the response of soil microbial activity to N and P addition in temperate forests of China.

## 5. Conclusions

By investigating responses of bacterial and fungal communities to nitrogen and phosphorus additions after four years in a temperate forest, we found that N addition decreased the bacterial diversity in O horizon, but tended to increase the bacterial diversity in A horizon. While P addition alone did not significantly change the bacterial diversity but it also influenced the bacterial diversity by interacting with N addition. Moreover, we observed significant and distinct shifts in the bacterial and fungal communities resulting from N and P additions, which were mainly due to the changes in soil pH and NO_3_^−^ contents. In general, N and P addition increased the relative abundance of typical copiotrophic bacterial taxa but reduced that of most oligotrophic groups. We also found that N and P additions changed most of the bacterial C and N transformation processes and several fungi guilds. Increased N deposition may lead to more C loss in O horizon, while P addition can alleviate or change the effects of increasing N deposition on the microbial functional potentials in O horizon and even also be a benefit for more C sequestration in A horizon. Our results of this study highlight the different responses of microorganisms to increasing N deposition and P addition between O and A horizon and predict the changes in forest carbon dynamics with N deposition in the future as well as the possible effects of P addition management.

## Figures and Tables

**Table 1:** The pairwise correlation analysis between soil microbial diversity index (Chao1, Observed species, Phylogenetic diversity and Shannon) and soil properties. Values in bold indicate significant correlations. * *p* < 0.05, ** *p* < 0.01 and *** *p* < 0.001.

	Soil Layer	Diversity Index	SOC	TN	TP	NO_3_^−^	NH_4_^+^	pH	MBC	MBN	MBP
Bacteria	O horizon	Chao1	0.556	0.301	−0.069	−0.684 *	0.784 **	0.770 **	0.281	0.601 *	0.41
		Observed species	0.526	0.316	0.000	−0.716 **	0.808 **	0.867 ***	0.261	0.634 *	0.501
		Phylogenetic diversity	0.521	0.232	0.153	−0.641 *	0.710 **	0.729 **	0.223	0.557	0.388
		Shannon	0.546	0.371	−0.008	−0.678 *	0.753 **	0.894 ***	0.266	0.601 *	0.538
	A horizon	Chao1	0.033	−0.075	0.245	0.297	−0.375	−0.759 **	0.353	0.115	0.148
		Observed species	0.196	0.037	0.338	0.525	−0.626 *	−0.626 *	0.401	0.098	0.334
		Phylogenetic diversity	−0.01	−0.196	0.209	0.375	−0.541	−0.552	0.243	−0.072	0.197
		Shannon	0.305	0.11	0.208	0.429	−0.634 *	−0.127	0.326	0.165	0.416
Fungi	O horizon	Chao1	−0.061	−0.276	0.584 *	−0.088	0.034	0.11	−0.184	−0.108	0.061
		Observed species	0.22	−0.081	0.477	−0.289	0.283	0.415	−0.045	0.107	0.097
		Phylogenetic diversity	0.422	0.1	0.391	−0.294	0.443	0.497	0.132	0.282	0.21
		Shannon	−0.021	−0.29	0.249	−0.28	−0.045	0.229	−0.166	−0.072	−0.31
	A horizon	Chao1	0.033	−0.075	0.245	0.297	−0.375	−0.759 **	0.353	0.115	0.148
		Observed species	0.196	0.037	0.338	0.525	−0.626 *	−0.626 *	0.401	0.098	0.334
		Phylogenetic diversity	−0.01	−0.196	0.209	0.375	−0.541	−0.552	0.243	−0.072	0.197
		Shannon	0.305	0.11	0.208	0.429	−0.634 *	−0.127	0.326	0.165	0.416

**Table 2:** Two-way PERMANOVA analysis (permutations = 999) estimated the effects of nitrogen addition (N), phosphorus addition (P) and N × P interaction on the soil microbial community structure. *p* values in bold indicate significant effects.

	Soil Layer	Effect	*R^2^*	*F*	*P*
Bacteria	O horizon	N	0.170	2.396	**0.020**
		P	0.099	1.391	0.158
		N × P	0.165	2.337	**0.023**
	A horizon	N	0.162	3.112	**0.028**
		P	0.259	4.979	**0.001**
		N × P	0.162	3.118	**0.032**
Fungi	O horizon	N	0.182	2.533	**0.003**
		P	0.134	1.855	**0.038**
		N × P	0.107	1.492	0.105
	A horizon	N	0.157	2.338	**0.004**
		P	0.161	2.393	**0.005**
		N × P	0.144	2.135	**0.017**

**Table 3:** The correlation between microbial community structure and soil variables as determined by the Mantel test (permutation = 999). The *r* and *P* indicate Pearson correlation coefficient and significance respectively, and *p* values in bold indicated significant correlations.

Variable	Bacteria				Fungi			
	O		A		O		A	
	*r*	*P*	*r*	*P*	*r*	*P*	*r*	*P*
SOC	0.2234	0.066	0.1408	0.151	0.1420	0.115	0.3204	**0.011**
TN	0.3859	**0.013**	0.1138	0.204	0.1238	0.212	0.1628	0.138
TP	−0.0627	0.637	0.3370	**0.029**	−0.1213	0.762	0.1978	0.115
NO_3_^−^	0.3291	**0.026**	0.7606	**0.001**	0.3815	**0.005**	0.2734	**0.042**
NH_4_^+^	0.4572	**0.007**	0.504	**0.001**	0.2456	0.058	0.1714	0.143
AN	0.3148	**0.018**	0.6085	**0.002**	0.386	**0.005**	0.2073	0.062
pH	0.7428	**0.001**	0.3829	**0.019**	0.3277	**0.009**	0.4481	**0.001**
MBC	0.0483	0.327	0.455	**0.005**	0.1533	0.16	0.1961	0.102
MBN	0.3642	**0.014**	0.0473	0.334	0.2502	**0.037**	0.0845	0.238
MBP	0.6281	**0.001**	0.5463	**0.004**	0.2686	**0.032**	0.2334	0.062
NO_3_^−^ + pH	0.3291	**0.016**	0.7658	**0.004**	0.3815	**0.003**	0.2774	**0.033**
All	0.1872	0.097	0.5723	**0.003**	0.2383	**0.045**	0.2155	0.061

## Supplementary Materials

The following are available online at https://www.mdpi.com/2076-2607/8/11/1828/s1, Table S1: Soil properties under different treatments in O and A horizons. C, N, P and NP indicate control (no addition), N addition, P addition and N coupled P addition; Table S2: Two-way ANOVA analysis estimated the effects of nitrogen addition (N), phosphorus addition (P) and N × P interaction on the soil properties; Table S3: Two-way ANOVA analysis estimated the effects of nitrogen addition (N), phosphorus addition (P) and N × P interaction on the soil microbial taxa; Table S4: The pairwise correlation analysis between soil bacterial taxa and soil properties; Table S5 The pairwise correlation analysis between soil fungal taxa and soil properties; Table S6 Two-way PERMANOVA analysis (permutations = 999) estimated the effects of nitrogen addition (N), phosphorus addition (P) and N × P interaction on the soil microbial functional composition., Table S7 Relative abundance in putative bacterial function groups under different fertilization treatments; Table S8 Relative abundance in putative fungal guilds under different fertilization treatments; Table S9 The numbers of Observed species from low abundance OTUs (no more than 5 observation counts in more than 53 thousand sequences for bacteria and no more than 3 observation counts in more than 35 thousand sequences for fungi).
