# Supplementary material for: Phosphorus Reduces Negative Effects of Nitrogen Addition on Soil Microbial Communities and Functions
Source: Microorganisms. 2020 Nov 20;8(11):1828. doi: 10.3390/microorganisms8111828 (PMC7699539; doi:10.3390/microorganisms8111828)
Supplement: Supplementary file 1 [file microorganisms-08-01828-s001.pdf]

Table S1 Soil properties under different treatments in O and A horizons. C, N, P and NP indicate control (no addition), N addition, P addition and N coupled P addition. All values are means ± standard errors. Different letters indicate significant differences among treatments for each soil horizon.

	O horizon				A horizon			
	C	N	P	NP	C	N	P	NP
pH	5.79±0.02a	4.96±0.08b	5.36±0.18b	5.22±0.24b	5.27±0.05a	5.17±0.06a	4.83±0.09b	5.14±0.03a
SOC (mg C g^{-1})	245.03±1.88a	203.79±7.83b	184.68±9.68b	233.91±13.44a	14.63±0.53b	17.56±1.43ab	15.14±2.90b	20.81±0.68a
TN (mg N g^{-1})	14.77±0.11a	13.39±0.16a	12.39±0.31a	14.39±1.83a	1.37±0.03b	1.52±0.28b	1.45±0.24b	2.04±0.14a
TP (mg P g^{-1})	1.12±0.06a	1.14±0.02a	1.24±0.05a	1.24±0.09a	0.57±0.01a	0.64±0.08a	0.72±0.15a	0.69±0.15a
NH_4^+-N (mg N kg^{-1})	7.25±0.43a	1.68±0.10c	3.05±0.32b	3.73±0.63b	5.00±0.60a	2.98±0.70b	3.72±0.43ab	2.67±0.34b
NO_3^--N (mg N kg^{-1})	75.83±8.98b	204.29±31.81a	127.66±17.71ab	187.27±48.34a	2.15±0.38b	7.11±0.93a	6.46±0.76a	8.10±1.40a
MBC (mg C kg^{-1})	4822.25±16.60a	4497.42±234.15ab	3859.20±265.57b	4558.98±350.92a	144.84±38.33b	232.78±40.08ab	373.01±121.71ab	445.64±137.12a
MBN (mg N kg^{-1})	880.13±8.94a	665.60±63.13b	609.91±62.50b	722.66±89.88ab	22.51±2.10bc	17.69±6.20c	35.84±9.92b	59.22±2.92a
MBP (mg P kg^{-1})	332.87±4.65a	234.12±28.49a	277.42±58.19a	339.20±86.81a	2.83±0.13c	7.00±0.28b	7.15±1.15b	12.55±1.80a
MBN:MBP	2.64±0.04ab	2.87±0.45a	2.23±0.30b	2.19±0.33b	7.96±0.39a	2.55±1.00c	4.95±0.60b	4.81±0.93b

Table S2 Two-way ANOVA analysis estimated the effects of nitrogen addition (N), phosphorus addition (P) and N×P interaction on the soil properties. *P* values in bold indicate significant effects.

	O horizon			A horizon		
	N	P	N×P	N	P	N×P
pH	**0.001**	0.35	**0.005**	**0.016**	**<0.001**	**<0.001**
SOC	0.474	**0.022**	**<0.001**	**0.002**	0.088	0.195
TN	0.576	0.238	**0.014**	0.12	**0.029**	0.086
TP	0.816	**0.012**	0.816	0.771	0.158	0.472
NH_4^+-N	**<0.001**	**0.002**	**<0.001**	**0.001**	**0.032**	0.152
NO_3^--N	**0.001**	0.353	0.087	**<0.001**	**0.001**	**0.016**
MBC	0.229	**0.014**	**0.007**	0.185	**0.004**	0.893
MBN	0.202	**0.019**	**0.002**	**0.03**	**<0.001**	**0.004**
MBP	0.571	0.451	**0.033**	**<0.001**	**<0.001**	0.354

Table S3 Two-way ANOVA analysis estimated the effects of nitrogen addition (N), phosphorus addition (P) and N×P interaction on the soil microbial taxa. *P* values in bold indicate significant effects.

	Taxonomic level	Dominant taxa	O horizon			A horizon		
			N	P	N×P	N	P	N×P
Bacteria	Phylum and subphylum	Proteobacteria	0.321	0.513	0.522	0.105	0.065	0.932
		Acidobacteria	0.362	0.791	0.153	0.078	**0.005**	**0.022**
		Actinobacteria	0.333	0.982	**0.037**	0.244	0.404	0.609
		Alphaproteobacteria	0.102	0.406	0.053	0.382	**0.006**	**0.003**
		Verrucomicrobia	0.88	0.384	0.709	0.203	**0.049**	0.086
		Chloroflexi	0.737	**0.024**	0.15	0.12	0.216	0.918
		Betaproteobacteria	0.164	0.813	0.11	**0.011**	**0.001**	**0.029**
		Planctomycetes	0.817	0.719	0.596	0.332	0.153	0.229
		Bacteroidetes	0.051	0.93	0.266	0.153	0.058	0.924
		Gammaproteobacteria	**<0.001**	0.336	**0.021**	0.343	0.258	0.981
		Deltaproteobacteria	0.087	0.568	0.088	0.558	0.455	0.246
		Gemmatimonadetes	0.061	0.12	0.891	**0.004**	**0.001**	**0.001**
		Nitrospirae	0.98	0.568	0.236	0.51	0.966	0.418
		Nitrospirae	0.98	0.568	0.236	0.51	0.966	0.418
Fungi	Phylum	Basidiomycota	0.602	0.669	0.923	0.890	0.603	0.553
		Ascomycota	0.306	0.971	0.963	0.578	0.612	0.992
		Mortierellomycota	0.116	0.078	0.227	0.342	0.107	0.068
		Cercozoa	**0.016**	0.623	0.176	0.219	0.754	0.618
		Chytridiomycota	**0.018**	0.646	0.813	0.698	0.473	0.385
		Rozellomycota	0.134	0.995	**0.028**	0.461	0.344	0.310
		Mucoromycota	0.054	0.292	0.518	0.370	**0.019**	0.491
		Kickxellomycota	0.485	0.142	**0.028**	0.783	0.956	0.226
		Glomeromycota	**0.006**	**0.001**	0.793	0.739	0.185	0.470
		Neocallimastigomycota	**0.021**	0.112	**0.025**	ns	ns	ns
	Class	Agaricomycetes	0.596	0.624	0.898	0.905	0.632	0.563
		Leotiomycetes	0.503	0.977	0.625	0.735	0.965	0.316
		Sordariomycetes	0.363	0.834	0.969	0.829	0.463	0.263
		Mortierellomycetes	0.119	0.079	0.236	0.341	0.107	0.068
		Eurotiomycetes	0.814	0.203	0.97	0.572	0.252	0.441
		Dothideomycetes	0.363	0.502	0.88	0.417	0.581	0.087
		Pezizomycetes	**0.008**	0.264	0.106	0.504	0.244	0.251
		Tremellomycetes	0.075	0.084	0.741	0.581	0.367	0.834

ns indicated null test because of zero OTU numbers existing in Neocallimastigomycota taxa.

Table S4 The pairwise correlation analysis between soil bacterial taxa and soil properties. Values in bold indicate significant correlations.

Soil layer	Bacterial taxa	SOC	TN	TP	NO3	NH4	pH	C:N	N:P
O horizon	Acidobacteria	0.453	0.280	-0.082	-0.173	0.505	0.473	0.386	0.254
	Actinobacteria	-0.502	-0.460	0.146	0.161	**-0.727****	-0.496	-0.233	-0.376
	Alphaproteobacteria	-0.479	-0.287	-0.159	0.497	**-0.599***	**-0.688***	-0.421	-0.147
	Verrucomicrobia	0.159	0.159	-0.446	0.084	0.174	-0.158	0.060	0.310
	Chloroflexi	0.200	0.061	0.406	-0.163	0.106	0.276	0.277	-0.112
	Betaproteobacteria	0.256	0.341	0.019	-0.489	**0.633***	**0.609***	-0.026	0.220
	Planctomycetes	-0.087	-0.328	0.199	0.051	-0.153	-0.037	0.264	-0.290
	Bacteroidetes	0.210	0.276	0.132	-0.562	**0.635***	**0.760****	-0.035	0.111
	Gammaproteobacteria	-0.316	-0.236	0.017	**0.826****	**-0.700***	**-0.869*****	-0.194	-0.195
	Deltaproteobacteria	0.484	0.522	-0.167	**-0.688***	**0.661***	**0.775****	0.127	0.432
	Gemmatimonadetes	-0.162	0.146	0.173	0.532	-0.428	-0.463	-0.407	-0.019
	Nitrospirae	0.259	0.386	-0.233	-0.160	0.405	0.139	-0.061	0.348
A horizon	Acidobacteria	-0.254	-0.217	-0.509	**-0.809****	**0.701***	0.552	-0.038	0.199
	Actinobacteria	-0.215	-0.156	0.153	-0.082	-0.023	-0.123	-0.058	-0.318
	Alphaproteobacteria	0.104	0.096	0.352	**0.654***	-0.424	**-0.797****	-0.052	-0.176
	Verrucomicrobia	0.450	0.423	0.481	**0.667***	-0.453	**-0.591***	-0.091	0.109
	Chloroflexi	0.426	0.448	**0.623***	**0.688***	**-0.621***	0.001	-0.084	-0.102
	Betaproteobacteria	-0.575	**-0.591***	**-0.748****	**-0.915*****	**0.751****	0.553	0.165	-0.012
	Planctomycetes	0.403	0.340	0.144	0.456	**-0.590***	-0.465	0.044	0.347
	Bacteroidetes	**-0.589***	**-0.643***	**-0.645***	**-0.687***	**0.614***	0.168	0.232	-0.192
	Gammaproteobacteria	-0.231	-0.345	**-0.635***	-0.534	0.313	0.024	0.271	0.147
	Deltaproteobacteria	-0.168	-0.112	0.012	0.350	-0.084	-0.330	-0.100	-0.151
	Gemmatimonadetes	-0.551	-0.479	-0.567	**-0.866*****	**0.748****	**0.647***	-0.025	-0.030
	Nitrospirae	-0.178	-0.166	0.105	0.250	-0.007	-0.029	0.030	-0.310

$^{*}P < 0.05$, $^{**}P < 0.01$, $^{***}P < 0.001$

Table S5 The pairwise correlation analysis between soil fungal taxa and soil properties. Values in bold indicate significant correlations.

Soil layer	Fungal taxa	SOC	TN	TP	NO3	NH4	pH	C:N	N:P
O horizon	Agaricomycetes	-0.036	0.226	-0.098	0.253	-0.099	-0.276	-0.322	0.165
	Leotiomycetes	-0.066	-0.315	0.091	-0.260	-0.029	0.135	0.266	-0.231
	Sordariomycetes	-0.007	-0.154	0.190	-0.337	0.160	0.450	0.161	-0.156
	Mortierellomycetes	0.427	0.348	-0.373	-0.421	**0.612***	**0.685***	0.229	0.425
	Eurotiomycetes	0.158	-0.163	-0.073	-0.165	0.190	0.115	0.441	-0.063
	Dothideomycetes	-0.194	-0.343	0.407	-0.272	0.083	0.347	0.088	-0.383
	Pezizomycetes	0.300	0.142	-0.071	**-0.762****	**0.780****	**0.795****	0.269	0.147
	Tremellomycetes	0.203	-0.178	-0.175	**-0.587***	0.457	0.464	0.512	0.002
A horizon	Agaricomycetes	0.059	0.156	-0.111	0.016	0.141	-0.431	-0.298	0.277
	Leotiomycetes	0.198	0.197	0.372	0.129	-0.181	0.384	0.009	-0.166
	Sordariomycetes	-0.336	-0.321	0.042	0.189	-0.198	-0.302	0.106	-0.372
	Mortierellomycetes	-0.289	-0.348	-0.218	-0.530	0.407	**0.661***	0.222	-0.145
	Eurotiomycetes	0.378	0.212	0.215	0.472	-0.555	-0.441	0.276	-0.038
	Dothideomycetes	0.245	0.054	0.548	0.525	**-0.627***	-0.282	0.427	-0.378
	Pezizomycetes	0.425	0.540	0.439	0.316	-0.339	0.093	-0.291	0.150
	Tremellomycetes	0.300	0.317	0.375	0.155	-0.212	-0.394	-0.101	-0.019

* $P < 0.05$, ** $P < 0.01$

Table S6 Two-way PERMANOVA analysis (permutations = 999) estimated the effects of nitrogen addition (N), phosphorus addition (P) and N×P interaction on the soil microbial functional composition.

	Soil layer	Effects	R^2	*F*	*P*
Bacteria	O horizon	N	0.032	0.370	0.791
		P	0.076	0.871	0.402
		N×P	0.197	2.265	0.105
	A horizon	N	0.152	3.057	0.076
		P	0.339	6.808	**0.008**
		N×P	0.111	2.240	0.141
Fungi	O horizon	N	0.120	1.469	0.233
		P	0.165	2.020	0.167
		N×P	0.060	0.728	0.454
	A horizon	N	0.180	2.237	0.111
		P	0.083	1.029	0.349
		N×P	0.091	1.134	0.314

Table S7 Relative abundance in putative bacterial function groups under different fertilization treatments. Values are mean ± standard deviation (n=3). Different letters behind values indicate significant differences (one-way ANOVA, *P* < 0.05, Duncan's multiple-range test) among different fertilization treatments.

Function categories	O horizon				A horizon			
	OC	ON	OP	ONP	AC	AN	AP	ANP
chemoheterotrophy	11.692±0.690a	12.700±0.767a	12.747±0.770a	11.850±0.414a	8.248±0.500b	9.197±0.269b	10.528±0.910a	9.186±0.597b
aerobic_chemoheterotrophy	10.809±0.673a	11.925±0.691a	11.891±0.804a	11.071±0.374a	7.763±0.501b	8.619±0.367b	9.934±0.954a	8.568±0.604b
cellulolysis	0.941±0.125a	1.291±0.139a	1.149±0.181a	0.996±0.467a	1.405±0.233c	2.743±0.497b	3.829±0.587a	3.223±0.602ab
nitrogen_fixation	1.339±0.179a	1.468±0.189a	1.543±0.089a	1.383±0.138a	2.683±0.072a	1.740±0.114b	1.568±0.174b	1.457±0.361b
ureolysis	0.294±0.051b	0.443±0.080a	0.336±0.070ab	0.378±0.022ab	1.341±0.058a	0.417±0.051b	0.281±0.074c	0.268±0.061c
hydrocarbon_degradation	0.265±0.022a	0.232±0.035a	0.249±0.039a	0.255±0.007a	0.331±0.013b	0.428±0.067a	0.451±0.057a	0.469±0.032a
methylotrophy	0.245±0.010a	0.215±0.028a	0.241±0.044a	0.246±0.014a	0.264±0.003b	0.323±0.045ab	0.319±0.068ab	0.364±0.046a
photoheterotrophy	0.249±0.014a	0.247±0.028a	0.273±0.024a	0.285±0.023a	0.201±0.035b	0.335±0.061a	0.288±0.078ab	0.319±0.071ab
methanotrophy	0.236±0.008a	0.207±0.035a	0.222±0.043a	0.230±0.012a	0.259±0.006b	0.316±0.043ab	0.314±0.072ab	0.358±0.043a
fermentation	0.461±0.048a	0.373±0.074ab	0.434±0.016ab	0.357±0.034b	0.148±0.009a	0.121±0.030a	0.128±0.025a	0.118±0.047a
aromatic_compound_degradation	0.206±0.054b	0.331±0.023a	0.177±0.048b	0.189±0.035b	0.208±0.050a	0.198±0.076a	0.197±0.037a	0.169±0.021a
photoautotrophy	0.254±0.016a	0.221±0.007a	0.238±0.018a	0.247±0.052a	0.168±0.027a	0.172±0.035a	0.109±0.012b	0.130±0.027ab
anoxygenic_photoautotrophy	0.204±0.002a	0.183±0.012a	0.206±0.021a	0.213±0.044a	0.144±0.021a	0.154±0.036a	0.088±0.014b	0.112±0.026ab
aromatic_hydrocarbon_degradation	0.021±0.012a	0.021±0.003a	0.021±0.003a	0.022±0.007a	0.071±0.009b	0.111±0.024a	0.136±0.019a	0.111±0.017a
aliphatic_non_methane_hydrocarbon_degradation	0.020±0.011a	0.020±0.004a	0.020±0.003a	0.022±0.007a	0.071±0.009b	0.111±0.024a	0.136±0.019a	0.111±0.017a
chitinolysis	0.053±0.007b	0.092±0.006a	0.076±0.032ab	0.085±0.008ab	0.005±0.006a	0.002±0.002a	0.002±0.001a	0.005±0.004a
anoxygenic_photoautotrophy_H2_oxidizing	0.012±0.005a	0.015±0.004a	0.015±0.002a	0.018±0.004a	0.067±0.004a	0.073±0.018a	0.034±0.011b	0.038±0.021b
iron_respiration	0.006±0.001b	0.006±0.001b	0.007±0.004b	0.015±0.004a	0.021±0.004a	0.022±0.011a	0.019±0.004a	0.023±0.005a
methanol_oxidation	0.009±0.003a	0.007±0.007a	0.019±0.009a	0.016±0.002a	0.005±0.003a	0.007±0.002a	0.005±0.005a	0.006±0.004a
chloroplasts	0.001±0.001a	0.004±0.004a	0.006±0.001a	0.019±0.029a	0.004±0.002b	0.013±0.003a	0.005±0.001b	0.009±0.006ab
chlorate_reducers	0.003±0.002a	0.001±0.002a	0.001±0.000a	0.002±0.003a	0.008±0.002b	0.018±0.009a	0.004±0.002b	0.002±0.001b
sulfate_respiration	0.005±0.004a	0.003±0.004a	0.002±0.003a	0.003±0.003a	0.002±0.002b	0.003±0.001b	0.003±0.001b	0.007±0.001a
manganese_reapiration	0.000±0.000a	0.000±0.001a	0.001±0.001a	0.002±0.003a	0.009±0.002a	0.009±0.008a	0.003±0.000a	0.003±0.002a

Table S8 Relative abundance in putative fungal guilds under different fertilization treatments. Values are mean ± standard deviation (n=3). Different letters behind values indicate significant differences (one-way ANOVA, *P* < 0.05, Duncan's multiple-range test) among different fertilization treatments.

Guilds	O horizon				A horizon			
	OC	ON	OP	ONP	AC	AN	AP	ANP
Ectomycorrhizal	16.206±1.983	26.370±15.730	28.864±18.017	38.316±18.414	13.088±5.626	41.430±20.452	35.517±24.051	42.939±12.930
Undefined Saprotroph	2.254±0.031	3.357±1.606	4.760±2.026	2.350±0.853	10.385±5.519	7.391±5.077	5.458±3.984	9.638±6.140
Plant Saprotroph-Wood Saprotroph	1.391±0.263	1.231±0.509	1.392±0.564	1.042±0.836	1.282±0.423	1.578±1.076	0.626±0.318	0.549±0.007
Animal Pathogen	2.992±0.207a	1.460±0.116b	0.952±0.112b	1.455±0.672b	0.194±0.037	0.283±0.192	0.245±0.085	0.153±0.028
Ectomycorrhizal-Fungal Parasite-Plant Pathogen-Wood Saprotroph	0.584±0.810	0.362±0.321	0.200±0.156	0.251±0.119	1.209±0.377	1.644±1.104	2.221±3.210	0.673±0.760
Undefined Saprotroph-Wood Saprotroph	1.291±0.441	1.113±0.432	1.732±0.631	1.187±0.614	0.157±0.135	0.147±0.081	0.241±0.138	0.249±0.102
Endophyte	1.144±0.194	0.764±0.062	1.170±0.553	0.925±0.596	0.586±0.106	0.375±0.158	0.471±0.284	0.556±0.124
Animal Pathogen-Undefined Saprotroph	1.778±0.501a	1.173±0.299ab	1.033±0.241b	0.828±0.233b	0.106±0.076	0.116±0.045	0.142±0.014	0.109±0.051
Endophyte-Plant Pathogen-Wood Saprotroph	1.367±0.108	1.136±0.918	0.718±0.413	0.905±0.491	0.057±0.033	0.094±0.027	0.085±0.041	0.152±0.136
Soil Saprotroph	0.170±0.045ab	0.235±0.081a	0.106±0.014b	0.220±0.086ab	0.936±0.988	0.880±0.612	0.497±0.384	1.360±1.448
Fungal Parasite	0.872±0.146	1.030±0.472	0.677±0.056	1.216±0.851	0.142±0.067	0.141±0.075	0.123±0.046	0.113±0.040
Ectomycorrhizal-Undefined Saprotroph	0.282±0.017b	0.044±0.054b	3.002±1.458a	0.089±0.010b	0.074±0.052	0.054±0.077	0.166±0.207	0.043±0.045
Ectomycorrhizal-Endophyte-Ericoid Mycorrhizal-Litter Saprotroph-Orchid Mycorrhizal	0.114±0.037	0.133±0.092	0.177±0.138	0.383±0.572	0.067±0.021	0.328±0.178	0.818±0.970	1.026±1.275
Animal Pathogen-Fungal Parasite-Undefined Saprotroph	0.320±0.168	0.213±0.189	0.396±0.092	0.349±0.065	0.166±0.017b	0.374±0.114ab	0.471±0.097ab	0.524±0.277a
Wood Saprotroph	0.445±0.021	0.290±0.157	0.833±0.708	0.368±0.174	0.029±0.007	0.178±0.139	0.043±0.009	0.498±0.764
Leaf Saprotroph	0.074±0.020	0.118±0.073	0.149±0.118	0.075±0.046	0.396±0.066	0.588±0.388	0.509±0.424	0.770±0.851
Plant Pathogen-Plant Saprotroph	0.157±0.045	0.128±0.069	0.175±0.064	0.136±0.042	0.086±0.056	0.267±0.092	0.106±0.071	1.009±1.593
Plant Pathogen	0.034±0.003	0.275±0.220	1.078±1.399	0.160±0.122	0.008±0.013	0.002±0.003	0.008±0.007	0.015±0.026
Dung Saprotroph	0.688±0.290a	0.121±0.044b	0.294±0.329ab	0.158±0.093b	0.004±0.004	0.014±0.018	0.008±0.008	0.002±0.002
Endophyte-Plant Pathogen-Undefined Saprotroph	0.395±0.039a	0.207±0.127b	0.131±0.096b	0.185±0.054b	0.010±0.007	0.013±0.018	0.007±0.006	0.011±0.010
Fungal Parasite-Undefined Saprotroph	0.067±0.027	0.084±0.069	0.105±0.085	0.046±0.016	0.055±0.037	0.144±0.106	0.193±0.183	0.183±0.141

Soil Saprotroph-Undefined Saprotroph	0.156±0.030	0.126±0.048	0.230±0.120	0.106±0.057	0.009±0.006	0.025±0.023	0.019±0.004	0.168±0.277
Ericoid Mycorrhizal	0.020±0.020	0.055±0.043	0.023±0.022	0.013±0.009	0.198±0.076	0.288±0.174	0.112±0.090	0.077±0.028
Endophyte-Litter Saprotroph-Wood Saprotroph	0.016±0.009b	0.313±0.230a	0.090±0.063ab	0.057±0.089b	0.003±0.003	0.051±0.059	0.009±0.010	0.095±0.160
Dung Saprotroph-Plant Saprotroph	0.032±0.019	0.028±0.031	0.125±0.129	0.128±0.021	0.052±0.010	0.080±0.037	0.032±0.017	0.087±0.049
Fungal Parasite-Plant Pathogen-Plant Saprotroph	0.037±0.022	0.013±0.018	0.134±0.183	0.076±0.023	0.033±0.021	0.050±0.026	0.047±0.026	0.140±0.200

Table S9 The numbers of Observed species from low abundance OTUs (no more than 5 observation counts in more than 53 thousand sequences for bacteria and no more than 3 observation counts in more than 35 thousand sequences for fungi). Values are mean ± standard deviation (n=3). Different letters behind values indicate significant differences (one-way ANOVA, $P < 0.05$, Duncan's multiple-range test) among different fertilization treatments. There were significant effects of N ($P = 0.005$) and N×P ($P = 0.003$) on rare bacterial species in O horizon soil, while there was a significant effect of N×P ($P = 0.031$) on rare fungal species in A horizon soil by two-way ANOVA analysis.

Soil layer	Treatments	Bacteria	Fungi
O horiozn	OC	618±35a	52±2a
	ON	382±19b	59±31a
	OP	462±58b	42±6a
	ONP	477±73b	45±14a
A horizon	AC	283±33a	10±6b
	AN	322±14a	19±7ab
	AP	294±60a	23±4a
	ANP	305±15a	15±5ab
